# Studies on the Interactions of Copper and Zinc Ions with β-Amyloid Peptides by a Surface Plasmon Resonance Biosensor

**DOI:** 10.3390/ijms130911832

**Published:** 2012-09-19

**Authors:** Fujun Yao, Ruiping Zhang, He Tian, Xiangjun Li

**Affiliations:** 1 College of Chemistry and Chemical Engineering, Graduate University, Chinese Academy of Sciences, Beijing, 100049, China; E-Mail: yaofuj09b@mails.gucas.ac.cn; 2 Institute of Materia Medica, Chinese Academy of Medical Sciences and Peking Union Medical College, Beijing 100050, China; E-Mail: tianhe@imm.ac.cn

**Keywords:** Alzheimer’s disease, β-amyloid peptide, surface plasmon resonance, metal ions

## Abstract

The aggregation of β-amyloid peptide (Aβ) into fibrils plays an important role in the pathogenesis of Alzheimer’s disease (AD). Metal ions including copper and zinc are closely connected to the precipitation and toxicity of Aβ. In this study, a surface plasmon resonance (SPR) biosensor was constructed to investigate the interactions between Aβ and metal ions. Aβ peptide was immobilized on the SPR chip surface through a preformed alkanethiol self-assembled monolayer (SAM). Our observations indicate that the immobilized Aβ undergoes a conformational change upon exposure to the metal ions. A difference in metal binding affinity between Aβ_1–28_ and Aβ_1–42_ was also detected. The results suggest that SPR is an effective method to characterize the interactions between Aβ and metal ions.

## 1. Introduction

Alzheimer’s disease (AD), which is characterized by irreversible and progressive neurodegeneration, is the dominant cause of dementia. The morphological hallmarks of AD are extracellular senile plaques and intracellular neurofibrillary tangles. The aggregated β-amyloid peptide (Aβ) containing 39–43 amino acid residues, which is generated from the amyloid precursor protein (APP) through the sequential cleavage by two enzymes, β-secretase and γ-secretase, is the principal component of the senile plaques. According to the amyloid cascade hypothesis, the aggregation of Aβ in AD leads to the formation of neurotoxic oligomers that are purportedly responsible for neuronal dysfunction and cell death [1]. Therefore, conditions that influence aggregation and the formation of oligomers are of great interest.

The aggregation of Aβ peptide is primarily affected by pH [2,3], peptide concentration [2], incubation time, membrane lipids [4] and temperature [5]. Moreover, a large body of evidence suggests that metal ions such as copper, zinc and iron may induce the aggregation of Aβ peptides, and these ligands may act as seeding factors in the formation of amyloid plaques [6–10]. In fact, elevated levels of zinc and copper have been found in amyloid plaques at concentrations reaching 1 mM and 400 μM, respectively [11]. These metal ions bind at the N-terminus (amino acids 1–16) and influence aggregation behavior. However, experimental evidence has shown that these metal ions in complex with Aβ peptides may have opposite functions with Zn accelerating aggregation, while Cu can reduce or accelerate aggregation [12–14]. Furthermore, it has been proposed that copper and iron mediate the production of reactive oxygen species (ROS) and oxidative stress [15,16]. It has been reported that reactive oxygen species (ROS) such as H_2_O_2_ are produced during the association of Aβ with Cu^2+^ through the reduction of Cu^2+^ to Cu^+^, which mediates cell toxicity [17]. Therefore, intensive efforts have been made to study the primary interaction of Aβ peptide with metal ions.

Surface Plasmon resonance (SPR) spectroscopy is capable of detecting a mass or conformational change above a metal surface. At a specific angle, the absorption of incident light by a thin metal film causes a collective oscillation of electrons in the film that launches an evanescent wave into the dielectric layer adjacent to the metal film. The propagation of the evanescent wave decays exponentially away from the metal film and is thus significantly perturbed by the adsorption of a species on the metal film or changes in the adlayer structure. Due to the advantages of real-time, label-free and direct detection of molecules in various media, SPR has become a popular and powerful technique to study molecular interactions. In recent years, SPR has been successfully employed to study biomolecular interactions related to AD [18,19]. Various aspects of Aβ oligomerization, fibril formation and extension and Aβ interactions with biomolecules have been investigated by SPR [20,21]. A custom-built flow-injection (FI) SPR instrument equipped with a bicell detector was constructed in our lab and used to investigate the interactions between Aβ_1–16_ and metal ions [22]. Immobilization of monomeric Aβ was performed using a self-assembled monolayer (SAM) of 11-mercaptoundecanoic acid (MUA) and amino coupling chemistry. To further test the feasibility of this biosensor, the interactions between metal ions and Aβ_1–28_ or the actual senile plaque component, Aβ_1–42_, were investigated.

## 2. Results and Discussion

### 2.1. Aβ Immobilization

**Figure 1:**
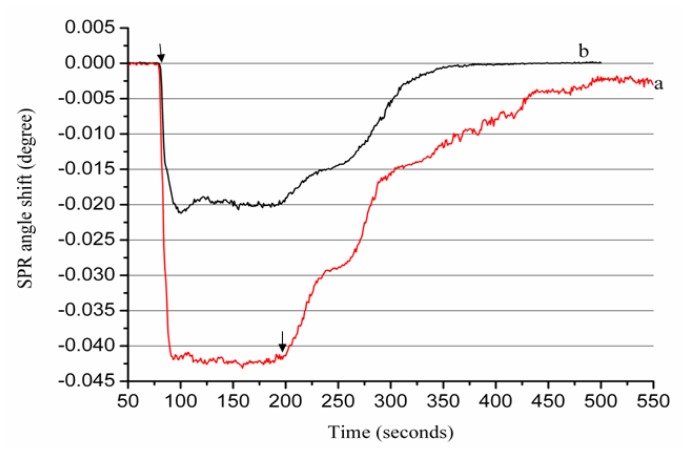
The sensorgram of a 200 μM Zn^2+^ ion solution flowing over the surface plasmon resonance (SPR) sensor chip with (**a**) and without (**b**) immobilized β-amyloid peptides (Aβ_1–28_) peptides. Arrows indicate Zn^2+^ ions starting to flow onto immobilized Aβ_1-28_ and when the flowing of metal ions ends, respectively.

The successful immobilization of the Aβ peptide on the sensor chip surface was tested by injecting a Zn^2+^ ion solution into the flow chamber and pumping it across the Aβ-immobilized sensing surface. In our experiment, the sample loop volume is 20 μL, the flowing rate is 10 μL/min, so that the Zn^2+^ ions start to flow onto the immobilized Aβ_1–28_ at about 80 s, the flowing of metal ions ends at about 200 s; the difference in the baseline SPR angles before and after the Zn^2+^ ion solution injection (termed as the SPR dip shift Δ*θ*) is approximately 0.00246° (Figure 1a), considering the sophisticated interaction between Aβ peptide and metal ions, the detection of the SPR dip shift Δ*θ* is selected at 550 s.

To confirm that the net change is induced by the metal ions, we conducted a control experiment in which Zn^2+^ ions flowed over an ethanolamine-blocked surface without the immobilization of Aβ peptide. Figure 1b shows that the SPR signal returned to its baseline within a short time. Additionally, the recovery of the original baseline in curve b suggests that the blocking process is efficient because it has been reported that alkanethiol SAM containing carboxylic acid can be used for SPR measurements of heavy metal ions [23]. Furthermore, the conversion of the negatively charged carboxyl groups on the MUA SAM to neutral amide groups during the peptide immobilization and blocking process could reduce the undesirable electrostatic attraction to metal ions in solution.

### 2.2. Real-Time Determination of Aβ_1–28_ Binding Zn^2+^ and Cu^2+^ by SPR

**Figure 2:**
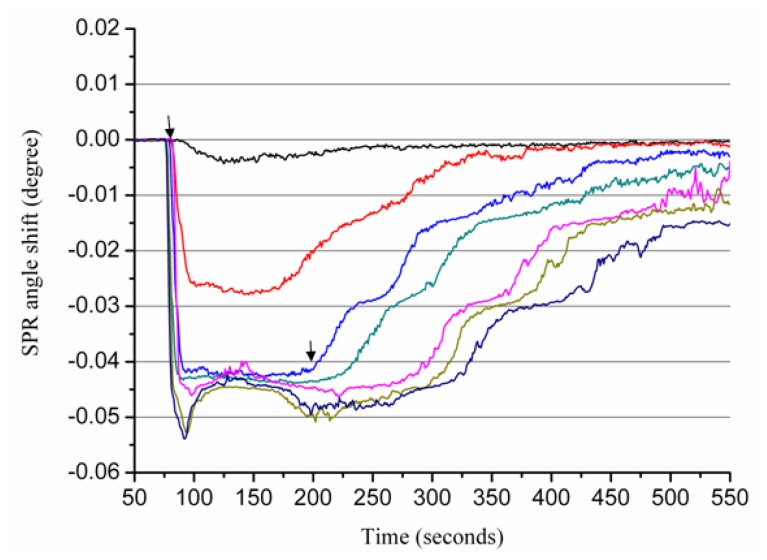
The sensorgrams for the interactions between Zn^2+^ ions and the Aβ_1–28_ peptides. Various concentrations (50, 100, 200, 300, 600, 700 and 800 μM, from top to bottom) of Zn^2+^ ions were injected onto the Aβ_1–28_ sensorchip. Arrows indicate Zn^2+^ ions starting to flow onto immobilized Aβ_1–28_ and when the flowing of metal ions ends, respectively.

Figure 2 shows the sensorgrams of various Zn^2+^ concentrations flowing over the Aβ_1–28_ sensor chip. In these experiments, EDTA (10 mM) was used to regenerate the surface. Therefore, a single chip can be used repeatedly for multiple samples. As shown in Figure 2, increasing Zn^2+^ concentrations results in a greater SPR dip shift. When 600 μM Zn^2+^ flowed over the Aβ_1–28_-covered SPR sensor, the metal ions caused a net change of 0.0075°, which can be attributed to the binding of Zn^2+^ ions to Aβ_1–28_ resulting in conformational changes of the peptide molecules.

**Figure 3:**
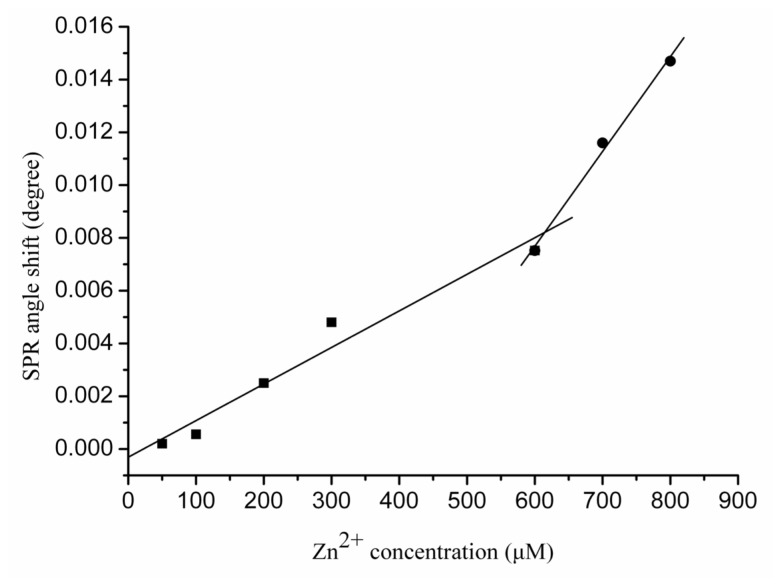
Calibration curves for the interactions between various Zn^2+^ concentrations with Aβ_1–28_. Each value represents the mean ± standard deviation of three separate injections.

The SPR angle shift is directly correlated with the Zn^2+^ concentration ([Zn^2+^]). In Figure 3, the calibration curve generated from the sensor response to a series of different Zn^2+^ concentrations is shown. The calibration curve contains two regions; the first is from 50 to 300 μM, and the second from 600 to 800 μM. Linear regression analysis of both regions yielded the following equations:

(1) $$\Delta \theta =-3.07\times 10^{-4}+1.39\times 10^{-5}\;[{\text{Zn}}^{2+}]\;R^{2}=0.95\;(\text{region }1)$$

(2) $$\Delta \theta =-1.39\times 10^{-2}+3.59\times 10^{-5}\;[{\text{Zn}}^{2+}]\;R^{2}=0.99\;(\text{region }2)$$

The slope of the linear regression in the second region is higher than that in the first region, which reveals that the SPR angle shift elicited by Zn^2+^ ions is greater with increasing Zn^2+^ ion concentration. These results suggest that the Zn^2+^-induced Aβ_1-28_ conformation change is concentration dependent. This finding is in agreement with a previous report indicating that Aβ undergoes a conformational change from a random coil to a regular secondary structure in the presence of Zn^2+^ ions and forms stable 1:1 and 1:2 (peptide/zinc) complexes [8]. A possible explanation for this phenomenon is that the Aβ_1–28_ binds Zn^2+^ intramolecularly at low concentrations. When the Zn^2+^ ion concentration is sufficiently high, the excess Zn^2+^ ions may begin to bind with Aβ_1–28_ intermolecularly, and the Zn^2+^ ions behave like a bridge connecting two adjacent Aβ_1–28_ molecules. Our finding is supported by previous studies that have shown that Zn^2+^ can coordinate to Aβ in an intra- and inter-peptide mode [24–26].

**Figure 4:**
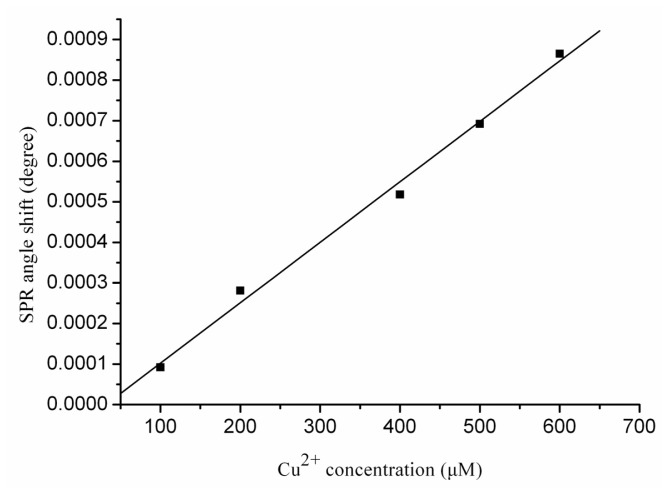
The calibration curve for the interactions between various Cu^2+^ concentrations with Aβ_1–28_. Each value represents the mean ± standard deviation of three separate injections.

A similar trend was not found for the interactions between Cu^2+^ and Aβ_1–28_. The calibration curve depicted in Figure 4 only contains one linear region ranging from 100 to 600 μM. The linear regression equation with a correlation coefficient of *R*^2^ = 0.99 suggests a linear relationship between the SPR angle shift and the Cu^2+^ concentration.

(3) $$\Delta \theta =-4.67\times 10^{-5}+1.49\times 10^{-6}\;[{\text{Cu}}^{2+}]\;R^{2}=0.99$$

Compared with Zn^2+^-Aβ complexes having stoichiometry ranging from 1:1 to 3:1 [27–29], most studies have demonstrated that the Aβ-peptide forms a 1:1 complex with Cu^2+^ [25,30,31]. NMR studies have demonstrated that Aβ_1–28_ forms a 1:1 complex with the Cu^2+^ ion via histidine residues [30]. We interpret these results to different coordination modes. Unlike Zn^2+^, which can bind to Aβ in an intra- and inter-peptide coordination mode, Cu^2+^ is primarily involved in intra-peptide binding in a fairly closed structure that protects the metal from further interactions. In addition, it has been reported that under physiological conditions, the coordination of the Cu^2+^ equivalent to the Aβ peptides leads to a mononuclear complex Cu_1_(Aβ)_1_ and is unlikely to form a Cu_2_(Aβ)_1_ complex [32].

### 2.3. Real-Time Determination of Aβ_1–42_ Binding to Zn^2+^ and Cu^2+^ by SPR

Aβ_1–40_ and Aβ_1–42_ are the most prevalent *in vivo* Aβ forms. In particular, Aβ_1–42_ has a high propensity to self-assemble and deposit in senile plaques and is highly toxic to neurons [33]. While great progress has been achieved on the coordination chemistry of Zn^2+^ and Cu^2+^ with truncated Aβ_1–16_ and Aβ_1–28_, it is important to understand whether these peptides, which are missing a large part of the hydrophobic *C*-terminal residues, coordinate metal ions differently than the full-length peptides. Therefore, to better understand the mechanisms of AD, we continued to study the interactions of Zn^2+^ and Cu^2+^ with Aβ_1–42_ using the FI-SPR biosensor.

**Figure 5:**
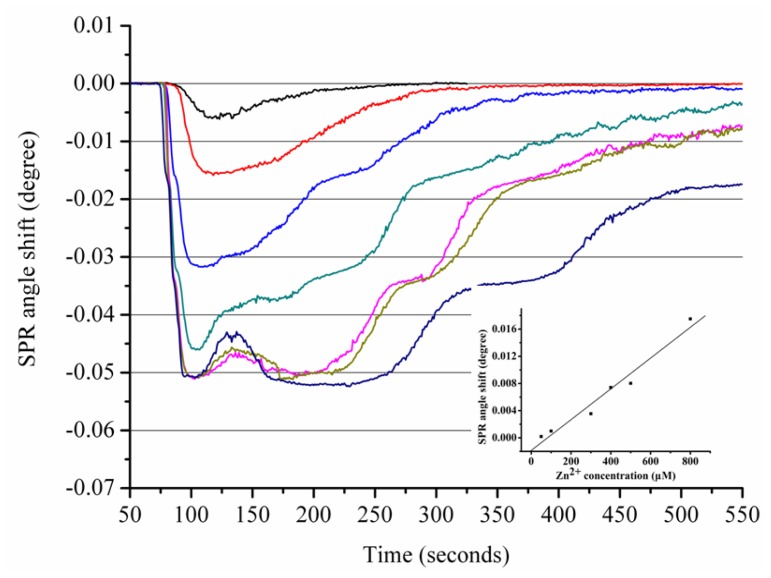
The sensorgram for the interactions between Zn^2+^ ions and Aβ_1–42_. Various concentrations (50, 100, 300, 400, 500 and 800 μM, from top to bottom) of Zn^2+^ ions were injected onto the Aβ_1–42_ sensorchip. The inset shows the calibration curve for the interactions between various concentrations of Zn^2+^ with Aβ_1–42_. Each value represents the mean ± standard deviation of three separate injections. Arrows indicate Zn^2+^ ions starting to flow onto immobilized Aβ_1–42_ and when the flow of metal ions ends, respectively.

Figure 5 depicts SPR sensorgrams obtained from the sensor response to various Zn^2+^ concentrations. In contrast to the calibration curve of the Zn^2+^ interactions with Aβ_1–28_ that contains two regions, the calibration plot of the Zn interactions with Aβ_1–42_ only contains one region; the linear regression equation is as follows:

(4) $$\Delta \theta =-1.86\times 10^{-3}+2.27\times 10^{-5}\;[{\text{Zn}}^{2+}]\;R^{2}=0.96$$

Aβ_1–42_ peptides have 14 additional residues in the hydrophobic tail compared to Aβ_1–28_, which makes Aβ_1–42_ more prone to aggregation than the truncated Aβ_1–28_. A possible explanation for the decreased stoichiometric binding of Aβ_1–42_ compared to Aβ_1–28_ may be the interference of the hydrophobic tail in Aβ_1–42_ with the Zn^2+^ binding site. Our data are in agreement with a previous report indicating that Zn and Cu form a monomeric complex with Aβ_1–42_ [34].

**Figure 6:**
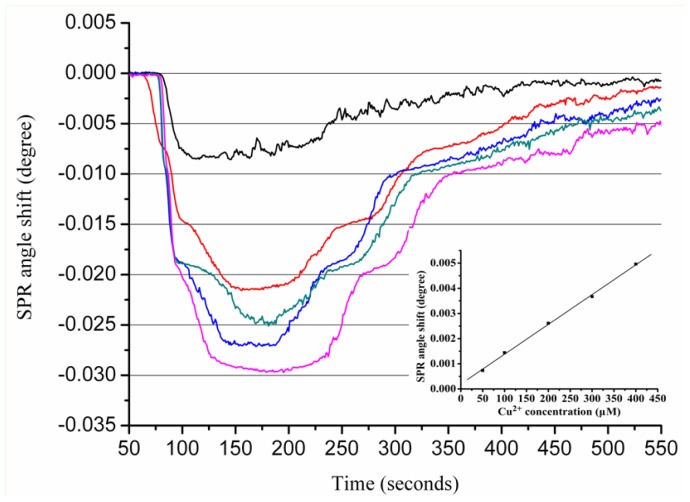
The sensorgram for the interactions between Cu^2+^ ions and Aβ_1–42_. Various concentrations (50, 100, 200, 300, and 400 μM, from top to bottom) of Cu^2+^ ions were injected onto the Aβ_1–42_ sensorchip. The inset shows the calibration curve for the interactions between various concentrations of Cu^2+^ with Aβ_1–42_. Each value represents the mean ± standard deviation of three separate injections. Arrows indicate Cu^2+^ ions starting to flow onto immobilized Aβ_1–28_ and when the flow of metal ions ends, respectively.

Similar results were obtained for the Cu-Aβ_1–42_ interaction. As shown in Figure 6, the SPR angle shift increased with an increase of Cu^2+^ bound to the Aβ_1–42_-immobilized sensor chip. The calibration curve generated from the sensor exposed to different concentrations of Cu^2+^ is shown in the Figure 6 inset. A strong correlation coefficient (*R*^2^ = 0.99) was obtained for the linear regression equation calculated using Cu^2+^ concentrations ranging from 50 to 400 μM.

## 3. Experimental Section

### 3.1. Chemicals

Human amyloid-β peptide (1–28) (Aβ_1–28_) and human amyloid-β peptide (1–42) (Aβ_1–42_) were obtained from GL Biochem Ltd. (Shanghai, China). 1-(3-Dimethylaminopropyl)-3-ethylcarbodiimide hydrochloride (EDC), *N*-hydroxysulfosuccinimide (NHS), 11-mercaptoundecanoic acid (MUA), ARC-grade dimethyl sulfoxide (DMSO, 99%) and ethanolamine were purchased from J & K Chemical Ltd. K_2_HPO_4_·3H_2_O, KH_2_PO_4_, NaCl, ZnCl_2_ and CuCl_2_ were all of AR grade and purchased from Beijing Chemical Reagent Co. (Beijing, China). Water with a resistivity of 18.25 MΩ·cm^−1^ was collected from a Millipore Simplicity 185 system. EDC (0.4 M) and NHS (0.1 M) were prepared in a pH 7.4 PBS buffer (10.0 mM K_2_HPO_4_·3H_2_O and KH_2_PO_4_ prepared in 1 mM NaCl). MUA (4 mM) was prepared in pure ethanol. Stock solutions of 2.0 mM ZnCl_2_ and CuCl_2_ were prepared in water and then diluted to the desired concentrations.

### 3.2. Preparation of Fresh Aβ Solution

Uniform and nonaggregated monomeric Aβ_1–28_ and Aβ_1–42_ peptides were prepared as previously described [35]. Briefly, 0.1 mM of the peptide was dissolved in DMSO, which disrupts the β-sheet structure and renders Aβ monomeric [36]. The freshly dissolved monomeric Aβ was diluted with PBS buffer (pH 7.4) to a concentration of 10 μM.

### 3.3. Gold Film Preparation

BK7 glass cover slides (Fisher) were cleaned with a piranha solution of 70% concentrated H_2_SO_4_ and 30% H_2_O_2_ (7:3, *v*/*v*) at 80 °C for 30 min. Upon cooling to room temperature, the glass slides were rinsed thoroughly with deionized water. After drying with N_2_, each glass slide was coated with a 2 nm chromium layer and then covered with 50 nm of gold film using a sputter coater (Model 108, Kert J. Lester Inc., Clariton, PA, USA).

### 3.4. Aβ Peptide Immobilization

**Figure 7:**
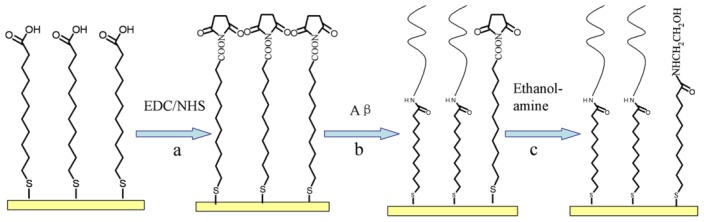
The procedure for the immobilization of Aβ. (**a**) The self-assembled monolayer (SAM) of MUA surface was activated with standard amine coupling chemistry using EDC/NHS; (**b**) The activated surface was covered with a fresh Aβ solution to form a bond between the amine group on the peptide and the carboxylic group on the MUA surface; (**c**) The remaining activated surface groups were blocked using ethanolamine.

Aβ peptide immobilization was achieved using covalent bonding mediated by a chemical reaction. The *N*-terminus of the Aβ peptide was reacted with the functional group of the SAM of MUA on the surface of the Au film (as depicted in Figure 7). Briefly, the MUA gold chip was treated with a mixture of 0.4 M EDC and 0.1 M NHS (1:1) for 3 h to ensure that the carboxyl group of the SAM reacted fully with the EDC and NHS. Then, a freshly prepared 10 μM Aβ solution in PBS (pH 7.4) was reacted with the NHS-activated surface for 2 h. Finally, ethanolamine (1 M, pH 8.5) was used to block the remaining activated surface groups. The resulting film was either used immediately or stored at 4 °C for future use.

### 3.5. SPR Apparatus

SPR measurements were conducted with a custom-built flow injection-SPR equipped with a bicell detector as previously described [22]. The SPR instrument recorded the reflected light on two photodetectors (A and B). The differential (A − B) and sum (A + B) signals were then detected by a PCI-1371 interface card (Advantech, Taiwan) controlled by the Labview program. The resonance angles from the biosensor were measured by the division of the differential and sum signals, (A − B)/(A + B). The inlet of the flow cell was connected to a six-port valve. For each measurement, the sample solution was injected into a 20-μL loop with a microsyringe (Hamilton) and subsequently delivered to the flow cell at a flow rate of 10 μL/min, using a syringe pump (KDS100, KD Scientific Inc., Holliston, MA, USA).

## 4. Conclusions

In the present study, a SPR-based analytical method was used to investigate the interactions between Aβ peptides and metal ions. The coordination of metal ions with the truncated Aβ_1–28_ and the full-length Aβ_1–42_ were compared. The conformational transition of Aβ induced by metal ion binding can be readily detected by this highly sensitive FI-SPR sensor equipped with a bicell detector. At physiological pH, Zn^2+^ demonstrates different binding affinity for Aβ_1–28_ and Aβ_1–42_, while Cu^2+^ exhibits similar interactions with both Aβ peptides. The interactions between Zn^2+^ and the Aβ-peptides in an intra- and inter-molecular mode have been previously validated. These studies complement other analytical methods and should help elucidate the role of metal ions during Aβ aggregation.
